# The Role and Effectiveness of Photodynamic Therapy on Patients With Actinic Keratosis: A Systematic Review and Meta-Analysis

**DOI:** 10.7759/cureus.26390

**Published:** 2022-06-28

**Authors:** George Mpourazanis, Wolfgang Konschake, Romanos Vogiatzis, Petros Papalexis, Vasiliki E Georgakopoulou, Georgios Ntritsos, Pagona Sklapani, Nikolaos Trakas

**Affiliations:** 1 Department of Obstetrics and Gynecology, General Hospital of Ioannina G. Hatzikosta, Ioannina, GRC; 2 Department of Dermatology, Ernst-Moritz-Arndt Medical University of Greifswald, Greifswald, DEU; 3 Department of Medicine, Faculty of Health Sciences, Aristotle University of Thessaloniki, Thessaloniki, GRC; 4 Unit of Endocrinology, First Department of Internal Medicine, Laiko General Hospital, National and Kapodistrian University of Athens, Athens, GRC; 5 Department of Pulmonology, Laiko General Hospital, Athens, GRC; 6 First Department of Pulmonology, Sismanogleio Hospital, Athens, GRC; 7 Department of Hygiene and Epidemiology, University of Ioannina Medical School, Ioannina, GRC; 8 Department of Informatics and Telecommunications, School of Informatics and Telecommunications, University of Ioannina, Arta, GRC; 9 Department of Cytology, Mitera-Hygeia Hospital, Athens, GRC; 10 Department of Biochemistry, Sismanogleio Hospital, Athens, GRC

**Keywords:** actinic keratosis, mal-pdt, bf-200 ala, daylight pdt, c-pdt, photodynamic therapy

## Abstract

Actinic keratoses (AKs) are the most common neoplastic lesions and are recognized as a precursor to squamous cell skin cancer. Photodynamic therapy (PDT) is a therapeutic option for multiple AKs in line with field cancerization. The aim of this study was to assess the effectiveness of PDT on patients with AKs using a meta-analysis, in order to evaluate the possible superiority of one treatment over the others. For this purpose, the PubMed, MEDLINE, Scopus, OVID, Science Direct, British Journal of Dermatology, Research Gate, and Embase databases were searched in March 2022. The search terms used were 'photodynamic therapy' and 'actinic keratosis'. We utilized the random-effects meta-analysis model to compare methyl aminolevulinate PDT (MAL-PDT) and the combination of a nanoscale-lipid vesicle formulation with the prodrug 5-aminolevulinic acid (BF-200 ALA) on a complete response (CR) of the lesions. Our meta-analysis indicated that the comparison of BF-200 ALA versus MAL-PDT showed marginally higher CRs than MAL-PDT.

## Introduction and background

According to scientific literature, actinic keratoses (AKs) are clinically observed lesions where dysplastic keratinocytes appear and which precancerous lesions of the skin have the potential to develop into squamous cell carcinomas (SCCs) in the future [1-2]. The risk of progression to an invasive tumor, such as SCCs, is estimated to be 0.60% after one year and 2.57% after four years [3]. It affects patients with fair skin, male sex, the elderly, and Fitzpatrick I or II phototype skin populations with chronic exposure to ultraviolet (UVB) radiation to areas such as the face, scalp, and back of the hands and causes induced mutations in the p53 tumor-suppressor gene [4-7]. The use of field-directed therapy is recommended to heal patients with AK [8]. Several treatments for AK exist and include surgical excision, cryotherapy, curettage, photodynamic therapy (PDT), and topical applications of components like 5-fluorouracil, imiquimod, ingenol mebutate, and diclofenac.

PDT is a modern and effective technique for AK and field cancerization [9-10]. The mechanism of PDT consists of using visible light that reacts with photosensitizing chemical compounds, 5-aminolevulinic acid (ALA), and methyl-aminolevulate (MAL). Both prodrugs enhance the formation of protoporphyrin IX (PpIX) and induce its accumulation due to cells’ altered metabolism. The interaction of visible light with ALA and MAL creates active oxygen species, which cause the apoptosis of skin cancer cells [11-13]. Daylight photodynamic therapy (DL-PDT) is used to treat AK and is more attractive, valuable, tolerable, and convenient. It is independent of light-emitting diode (LED) light compared to conventional photodynamic therapy (C-PDT) because it involves exposure to direct daylight [14-17].

The scope of this study was to compare the effectiveness of BF-200 ALA with MAL-PDT on patients with AK. More specifically, we compared both therapies using meta-analysis based on scientific literature in order to find out if these therapies show a significant statistical difference.

## Review

Materials and methods

Literature Search and Data Extraction

We searched the following databases: PubMed, MEDLINE, CINAHL, Scopus, OVID, Embase, Science Direct, Research Gate, and British Journal of Dermatology. Studies were included from the last two decades. The search terms were "photodynamic therapy AND actinic keratosis." We included randomized clinical trials, randomized prospective studies, intraindividual randomized trials, and randomized comparison studies involving patients with actinic keratosis and photodynamic therapy such as MAL-PDT therapy, BF-200 ALA, C-PDT, DL-PDT, ablative fractional laser resurfacing (AFXL)-PDT, and MAL-DL-PDT.

Data extraction was undertaken separately for each intervention. All relevant information was extracted for each study: first author/year of publication; type of study; type of therapy/number of patients; age; skin phototype, number of lesions treated; and results. The search was also limited to English-language articles. The reference lists of the included articles have also been searched for additional studies.

Data Synthesis and Analysis

The data evaluating the primary and secondary outcomes for each trial was expressed as a relative risk ratio (RR) with a 95% confidence interval (CI). More than two studies were available for the same treatment comparison on lesion complete remission (CR), we summarized RRs and 95% CIs using fixed-effects and random-effects meta-analysis models [18]. The Cochran's Q test and the I2 statistic were used to assess between-study heterogeneity [19]. The presence of small-study effects was assessed using Egger’s regression asymmetry test if at least 10 RCTs reported a specific comparison [20]. The analysis was performed in STATA 14 (StataCorp. 2015. Stata Statistical Software: Release 14. College Station, TX: StataCorp LP). The p-values were all two-tailed.

Results

**Figure 1:**
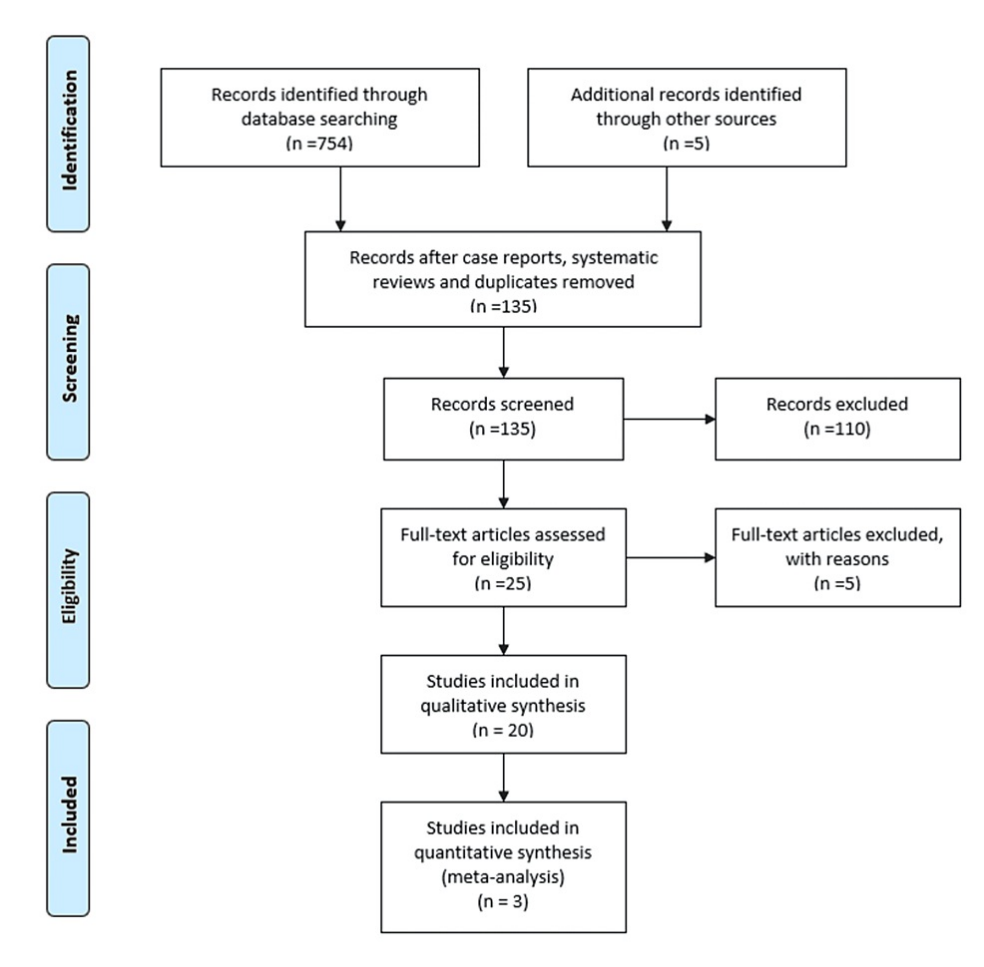
Selection process of the included studies for the systematic review and meta-analysis

The study has been designed and the results have been reported based on the Preferred Reporting Items for Systematic Reviews and Meta-Analyses (PRISMA) statement (Figure 1) [21].

Our literature search yielded 759 studies. After excluding case reports, systematic reviews, duplicate studies, studies that did not present any relevant data or did not conform to the inclusion or exclusion criteria, and studies that referred to diseases other than actinic keratosis and treatment other than photodynamic therapy, we ended up with 20 studies.

**Figure 2:**
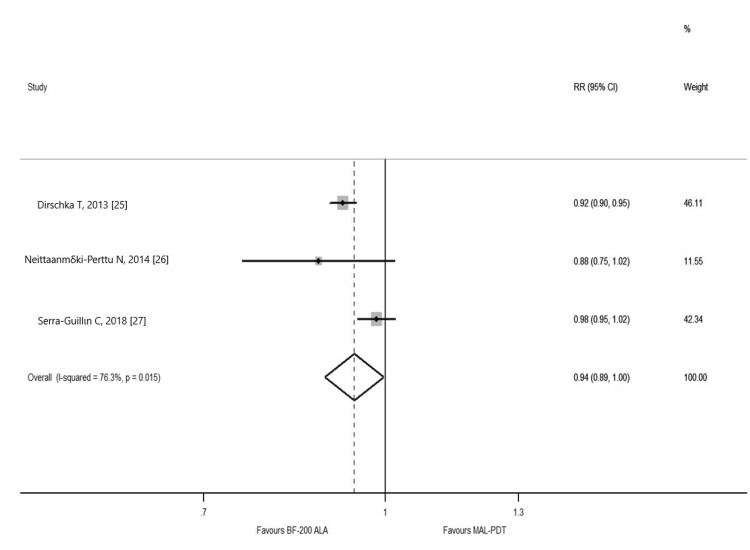
Forest plot for the comparison of CR for BF-200 ALA vs. MAL-PDT BF-200 ALA: a combination of a nanoscale-lipid vesicle formulation with the prodrug 5-aminolevulinic acid; MAL-PDT: methyl aminolevulinate photodynamic therapy [25-27]

We performed one meta-analysis of randomized controlled clinical trials (RCTs) based on comparisons on CR (Figure 2). Overall, three RCTs comparing the reported lesion CRs achieved by MAL-PDT and BF-200 ALA provided sufficient data to perform a meta-analysis. We also performed a random-effects meta-analysis model to compare the effectiveness of MAL-PDT and BF-200 ALA on lesion CRs. BF-200 ALA showed marginally higher CRs than MAL-PDT did (Figure 2; N = 3 RCTs; RR = 0.94; p-value = 0.042). Substantial between-study heterogeneity was observed (I2 = 76.3%). As we included fewer than 10 studies, we did not assess the presence of small-study effects. In our study, three RCTs were used for meta-analysis to compare statistically both therapies. In total, four RCTs compared the reported lesions CRs achieved by DL-PDT with those achieved by C-PDT [14,22-24]. Additionally, three RCTs compared the reported lesion CRs achieved by MAL-PDT with those achieved by BF-200 ALA [25-27]. The meta-analysis revealed that BF-200 ALA showed marginally higher CR than MAL-PDT.

Discussion

PDT is widely used in clinical practice to cure AK and many other diseases. Between the two prodrugs, BF-200 ALA and MAL, the only licensed form is MAL-PDT [28]. Several RCT studies have shown the effectiveness, complete response to treated lesions, and cosmetic outcomes using MAL-PDT and BF-200 ALA. BF-200 ALA has been shown to be more tolerable for patients. Furthermore, it produces better cosmetic results, causes less pain, and has a higher overall response than MAL-PDT [25-27,29]. Another study that used BF-200 ALA gel and MAL cream to treat AK lesions for 12 weeks demonstrated a recurrence rate of 19.9% for lesions treated with BF-200 ALA, and 31.6% for lesions treated with MAL [29].

Post-marketing surveillance trials with a total sample size of n = 4109 observed that the lesion-specific clearance rate for PDT was 74% (95% confidence interval (CI) 56-87%) compared to other treatments such as ingenol mebutate gel, diclofenac sodium, and imiquimod cream [30]. A study using MAL and BF-200 ALA indicated that BF-200 ALA had a complete clearance rate of 79.7% and MAL 73.5% [31]. A randomized double-blind trial compared both ALA-PDT and MAL-PDT for extensive scalp AK and showed that these treatments result in a significant reduction in scalp AK, but there is no significant difference in efficacy [32].

C-PDT is known worldwide as an effective treatment for multiple AKs and in large areas with high response rates and excellent cosmetics [33-34]. Limiting factors of the C-PDT in clinical practice display side effects such as burning, prickling sensations, neuropathic pain, pain during treatment, procedure prolonged clinic visits, and the need for artificial light sources. DL-PDT according to studies performed in Scandinavia, Australia, and Southern Europe has shown to provide similar AK clearance rates to C-PDT. Furthermore, the pain was almost similar to the pain caused by C-PDT regardless of the weather conditions [35-37]. A recent Chinese meta-analysis included six RCTs and a total of 369 patients with AKs treated with DL-PDT and C-PDT and MAL patients with AK that received D-PDT were associated with a significantly lower maximal pain score and fewer incidence of adverse events than those that received C-PDT with red light. In conclusion, D-PDT is better tolerated than C-PDT in patients with AK [38]. Overall, based on published scientific literature, it appears that the comparison of C-PDT and DL-PDT has no statistically significant results.

The general characteristics of the studies are summarized in Table 1.

**Table 1:** Characteristics of the included studies *BF200-ALA: a combination of a nanoscale-lipid vesicle formulation with the prodrug 5-aminolevulinic acid; CR: complete response; PDT: photodynamic therapy; AFXL-PDT: ablative fractional laser resurfacing photodynamic therapy; C-PDT: conventional photodynamic therapy; DL: daylight photodynamic therapy; MAL-PDT: methyl aminolevulinate photodynamic therapy

First author/Year of publication	Type of study	Type of therapy/Number of patients	Age (years)	Skin Phototype	Number of treated lesions	Results (CR%)
Rodringo C, 2019 [39]	Intrapatient randomized trial	MAL-PDT and DL-PDT/31	76.8 ± 8.8	I, II, III	166.3	CR for MAL: 80.7 and for DL: 85.6
Heerfordt IM, 2019 [40]	Randomized controlled trial	DL-PDT/25	54-84	I, II	DL-PDT: 400	CR for DL-PDT: 86
Seo JW, 2019 [41]	Single-blinded randomized comparative prospective trial	AFL-PDT/60	Not mentioned	I, II, III	AFL-PDT:121	CR: 79.5
Vrani F, 2019 [42]	Randomized intraindividual comparison study	AFXL-PDT and C-PDT/ 42	>18	I, II, III	AFXL-PDT: 90.5 and C-PDT: 92.8	CR AFXL 47.2 and C-PDT 52.3
Cantisani C, 2018 [43]	Randomized controlled trial	MAL-DL-PDT/93	Mean age 72	Not mentioned	43	CR for MAL-DL-PDT: 90
Wiegell SR, 2019 [44]	Randomized controlled trial	MAL-DL-PDT/25	Not mentioned	Not mentioned	75.8	CR for MAL-DL-PDT: 75.8
Miola AC, 2018 [45]	Randomized controlled trial	MAL-PDT:/36	>18	I, II, III	MAL-PDT: 67	CR for MAL-PDT 67
Zhu L, 2018 [22]	Randomized and prospective study	DL-PDT/30, C-PDT/30	Mean age 74.6	I, II, III	C-PDT:76.7, DL-PDT:66.7	CR for DL-PDT: 95.5 and for C-PDT:96.8
Dirschka T, 2018 [29]	Randomized intra-individual non-inferiority phase III study	DL(MAL)-PDT/52, BF-200 ALA/52	18-85	I, II, III, IV, V	BF-200 ALA: 6.4 ± 2.2 and DL-PDT: 6.4 ± 2.2	CR for DL-PDT: 79.8 and BF-200 ALA: 76.5
Serra-Guillén C, 2018 [27]	Randomized intraindividual comparative study	MAL-PDT/25, BF-200 ALA/25	Mean age 72.2	II	MAL-PDT:600 and BF200 ALA:604	CR for MAL-PDT:56 and BF-200 ALA: 62
Sotiriou E, 2018 [23]	Randomized intra-individual comparative analysis	DL-PDT/23, C-PDT/23	59-84	I, II, III	C-PDT:217, DL-PDT:236	CR for DL-PDT: 78 and C-PDT:80.6
Lacour JP, 2015 [24]	Randomised investigator-blinded controlled phase III study	DL-PDT and C-PDT/108	Mean age 47.91	I, II, III, IV	C-PDT:960, DL-PDT:957	CR for DL-PDT: 74 and C-PDT: 70
Song HS, 2015 [46]	Randomized controlled trial	AFXL-PDT/24 and C-PDT.34	Not mentioned	I, II, III	AFXL-PDT:25 and C-PDT:22	CR for AFXL-PDT:71.4 and C-PDT: 64.2
Rubel DM, 2014 [14]	Randomized controlled trial	DL-PDT and C-PDT/100	Mean age 42-90	I, II, III	C-PDT: 1372, DL-PDT: 1379	CR for DL-PDT: 89.2 and C-PDT: 92.8
Neittaanmäki-Perttu N, 2014 [26]	Randomized double-blinded nonsponsored prospective study	MAL-PDT and BF-200 ALA/13	Not mentioned	I, II, III	MAL-PDT:93, BF-200 ALA: 84	CR for MAL-PDT: 74.2 and BF-200 ALA: 84.5
Helsing P, 2013 [47]	Randomized half-side comparative trial	AFXL PDT/8 and only AFXL /0	55-74	II, III	AFXL-PDT:335, AFXL alone: 345	CR for AFXL-PDT: 73
Dirschka T, 2013 [25]	6 and 12 months follow-up of two prospective randomized controlled phase III trials	MAL-PDT/240 BF-200 ALA/241	50-87	I, II	BF-200 ALA: 1359 and MAL-PDT: 1295	CR for MAL-PDT:36 and BF-200 ALA: 47
Scola N, 2012 [48]	Randomized half-side comparative study	PDT and AFXL-PDT/32	55-84	Not mentioned	Not mentioned	Not mentioned
Szeimies RM, 2010 [49]	Prospective randomized double-blind placebo-controlled phase III study	MAL-PDT/41 BF-200 ALA:/81	18 -85	I, II, III, IV, V, VI	BF-200 ALA: 463, MAL-PDT: 225	CR for MAL-PDT: 81 and BF-200 ALA: 64

This review has some limitations. The types of studies weren’t similar in the meta-analysis we performed. Because AK is a chronic disease, the follow-up period could be considered relatively short. There weren’t many studies using the same treatment protocol for the AKs. Common PDT treatment variations include topical sensitizer, incubation time, light source, exposure time, and lesion preparation. Moreover, some of the studies analyzed were blinded, and thus the other non-blinded studies were susceptible to bias. Another limitation is that all studies had PDT as a common treatment, but not all patients continued the therapy.

## Conclusions

Studies have shown that all treatments (DL-PDT, C-PDT, BF-200 ALA, MAL-PDT) are effective in patients with AK and can be clinically applied. In this systematic review, our meta-analysis showed that the comparison of BF-200 ALA versus MAL-PDT yielded a marginally higher CR than MAL-PDT. More clinical trials are needed in order to strengthen our results.
